# Combined PD-1, BRAF and MEK inhibition in BRAF^V600E^ colorectal cancer: a phase 2 trial

**DOI:** 10.1038/s41591-022-02181-8

**Published:** 2023-01-26

**Authors:** Jun Tian, Jonathan H. Chen, Sherry X. Chao, Karin Pelka, Marios Giannakis, Julian Hess, Kelly Burke, Vjola Jorgji, Princy Sindurakar, Jonathan Braverman, Arnav Mehta, Tomonori Oka, Mei Huang, David Lieb, Maxwell Spurrell, Jill N. Allen, Thomas A. Abrams, Jeffrey W. Clark, Andrea C. Enzinger, Peter C. Enzinger, Samuel J. Klempner, Nadine J. McCleary, Jeffrey A. Meyerhardt, David P. Ryan, Matthew B. Yurgelun, Katie Kanter, Emily E. Van Seventer, Islam Baiev, Gary Chi, Joy Jarnagin, William B. Bradford, Edmond Wong, Alexa G. Michel, Isobel J. Fetter, Giulia Siravegna, Angelo J. Gemma, Arlene Sharpe, Shadmehr Demehri, Rebecca Leary, Catarina D. Campbell, Omer Yilmaz, Gad A. Getz, Aparna R. Parikh, Nir Hacohen, Ryan B. Corcoran

**Affiliations:** 1 Massachusetts General Hospital Cancer Center and Harvard Medical School, Boston, MA USA; 2 The Broad Institute of Massachusetts Institute of Technology and Harvard, Cambridge, MA USA; 3 Gladstone-UCSF Institute of Genomic Immunology, Gladstone Institutes Department of Microbiology and Immunology, UCSF, San Francisco, CA USA; 4 Dana Farber Cancer Institute and Harvard Medical School, Boston, MA USA; 5 The Koch Institute, Massachusetts Institute of Technology, Cambridge, MA USA; 6 Department of Immunology, Blavatnik Institute, Harvard Medical School, Boston, MA USA; 7 Novartis Institute for Biomedical Research, Cambridge, MA USA

**Keywords:** Cancer immunotherapy, Colorectal cancer

## Abstract

While BRAF inhibitor combinations with EGFR and/or MEK inhibitors have improved clinical efficacy in BRAF^V600E^ colorectal cancer (CRC), response rates remain low and lack durability. Preclinical data suggest that BRAF/MAPK pathway inhibition may augment the tumor immune response. We performed a proof-of-concept single-arm phase 2 clinical trial of combined PD-1, BRAF and MEK inhibition with sparatlizumab (PDR001), dabrafenib and trametinib in 37 patients with BRAF^V600E^ CRC. The primary end point was overall response rate, and the secondary end points were progression-free survival, disease control rate, duration of response and overall survival. The study met its primary end point with a confirmed response rate (24.3% in all patients; 25% in microsatellite stable patients) and durability that were favorable relative to historical controls of BRAF-targeted combinations alone. Single-cell RNA sequencing of 23 paired pretreatment and day 15 on-treatment tumor biopsies revealed greater induction of tumor cell-intrinsic immune programs and more complete MAPK inhibition in patients with better clinical outcome. Immune program induction in matched patient-derived organoids correlated with the degree of MAPK inhibition. These data suggest a potential tumor cell-intrinsic mechanism of cooperativity between MAPK inhibition and immune response, warranting further clinical evaluation of optimized targeted and immune combinations in CRC. ClinicalTrials.gov registration: NCT03668431.

## Abstract (web summary)

Patients with BRAF^V600E^-mutated colorectal cancer have encouraging overall response rates to inhibition of PD-1, BRAF and MEK, with translational analyses suggesting that induction of tumor-intrinsic programs and immune programs contributes to improved outcomes via MAPK inhibition.

## Main

BRAF^V600E^ mutations occur in ~10% of colorectal cancer (CRC), driving constitutive activation of MAPK signaling. Patients with BRAF^V600E^ CRC have unfavorable prognosis and respond poorly to standard therapies, with a median overall survival (OS) half that of BRAF wild-type CRC^1,2^. While BRAF inhibitors (BRAFis), including vemurafenib and dabrafenib, are highly effective in BRAF^V600E^ melanoma (~60–80% response rate)^3,4^, the response rate of BRAFi monotherapy in BRAF^V600E^ CRC is only 0–5%^5,6^. Previous work by our group and others identified robust adaptive feedback networks in CRC leading to rapid reactivation of MAPK signaling following BRAF inhibition, including EGFR, which acts as the dominant driver in many cases^7–9^. These data led to clinical trials of BRAFi-based therapeutic combinations designed to mitigate MAPK feedback reactivation and produce sustained MAPK suppression, yielding increased response rates in BRAF^V600E^ CRC. Specifically, combinations of BRAFi and EGFR inhibitor (EGFRi), BRAFi and MEK inhibitor (MEKi), and BRAFi, EGFRi and MEKi have been explored clinically^10–12^. Recently, the Food and Drug Administration approved the combination of the BRAFi encorafenib plus the anti-EGFR antibody cetuximab in BRAF^V600E^ CRC^10,12^. However, confirmed objective response rates (cORRs) to this regimen are only 20% and clinical benefit is not durable, with a median progression-free survival (PFS) of only 4.3 months. Thus, new effective therapies for this disease are critically needed.

Immune checkpoint blockade (ICB), particularly agents that block the programmed death (PD)-1 pathway, has revolutionized the treatment of many cancers with the potential for long-term durable responses^13,14^. CRC has generally responded poorly to ICB, with the exception of the ~4% of metastatic CRC with microsatellite instability (MSI)/mismatch repair deficiency, in which response rates are ~40%, likely due to increased neoantigen load^15^. Conversely, response rates in metastatic microsatellite stable (MSS) CRC are near 0%^16^. Thus, approaches to increase the immune responsiveness of MSS CRC represent a key unmet clinical need.

Interestingly, ~15–20% of BRAF^V600E^ metastatic CRC harbors MSI^17^, and these patients also showed better and more durable responses to BRAF/MAPK pathway inhibition than patients who are MSS in prior clinical trials of BRAF/MEK/EGFR inhibition, despite receiving targeted BRAF-directed therapy only. Indeed, approximately one third of patients with BRAF^V600E^ MSI exhibited durable response and/or disease control lasting >1 year in our previous clinical trial^12^. Conversely, no patients who are MSS remained on study >1 year. Moreover, the lone patient achieving complete response (CR) in our initial study evaluating combined BRAF/MEK inhibition with dabrafenib and trametinib (DT)—which produced a cORR of 7% (12% unconfirmed ORR)—also had MSI and remained in an ongoing durable CR for >5 years^11^. Given that durable responses were restricted to patients with MSI, these data suggest the possibility that BRAF pathway inhibition may enhance immune response in BRAF^V600E^ CRC.

Preclinical models suggest that combining MAPK inhibition and ICB could enhance antitumor efficacy in BRAF and KRAS mutant cancers^18–20^. Several potential mechanisms of cooperativity have been proposed, including possible immune priming of the tumor microenvironment by a direct effect of BRAFi and/or MEKi on nontumor cells, such as antigen-specific and activated CD8^+^ T cells and expanded memory T cells and T cell clonotypes. The potential for tumor-intrinsic effects by MAPK inhibition contributing to the immune response has also been proposed^19^. However, a definitive mechanism for this potential cooperativity has not been established. Moreover, clinical trials in BRAF^V600E^ melanoma demonstrated promising efficacy and long-lasting antitumor responses with the combined inhibition of BRAF/MEK and PD-1/PD-L1 pathways^21–24^.

Based on these observations, we investigated the potential cooperativity of BRAF/MAPK pathway inhibition and ICB in BRAF^V600E^ CRC. We studied paired biopsies from previous clinical trials of patients with BRAF^V600E^ CRC treated with BRAF-targeted combinations and preclinical immune-competent mouse models of BRAF^V600E^ CRC. We also performed the first clinical trial, to our knowledge, of BRAF-targeted therapy combined with ICB specifically in patients with BRAF^V600E^ CRC, evaluating the efficacy of combined BRAF, MEKi and PD-1 inhibition. All patients underwent paired pretreatment and day 15 on-treatment tumor biopsies, which were evaluated by single-cell RNA sequencing (scRNAseq) to elucidate potential mechanisms of cooperativity.

## Results

### MAPK inhibition enhances immune response in BRAF^V600E^ CRC

**Fig. 1:**
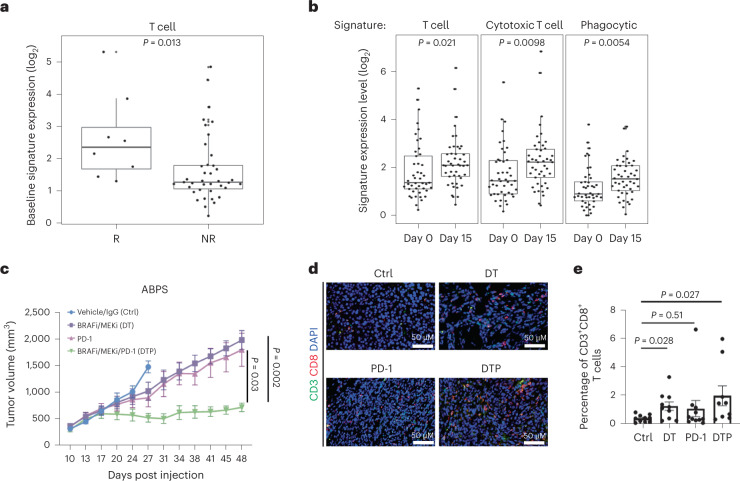
MAPK pathway inhibition enhances immune response in BRAF^V600E^ CRC. **a**, Tumor baseline T cell signature expression levels in confirmed responders (R) (*n* = 8) and nonresponders (NR) (*n* = 39) from a clinical trial of dabrafenib/trametinib/panitumumab in patients with BRAF^V600E^ CRC (DTP treatment arm only, two-tailed Wilcoxon rank sum tests). **b**, Levels of immune signatures (T cell, cytotoxic T cell and phagocytic) in 45 paired day 1 and day 15 biopsies from a clinical trial of dabrafenib/trametinib/panitumumab (all DTP, DP and TP treatment arms, two-tailed Wilcoxon rank sum tests). **a**,**b**, The box plots show the median, first and third quartiles (Q1 and Q3) (25th and 75th percentiles) of the data. The upper and lower whiskers extend to the minimum and maximum values no further than 1.5× the interquartile range, respectively; outliers are plotted individually. **c**, Tumor volume of C57BL/6 mice bearing ABPS tumors treated with vehicle/immunoglobulin G control (*n* = 11), DT (*n* = 12), PD-1 (*n* = 11) and DTP (*n* = 12; two-tailed Wilcoxon rank sum test, data are presented as mean values ± s.e.m.). **d**, Representative images of CD3^+^CD8^+^ T cells in ABPS tumors. **e**, Percentage of CD3^+^CD8^+^ T cells in ABPS tumors across the groups control (*n* = 10), DT (*n* = 10), PD-1 (*n* = 11) and DTP (*n* = 9; two-tailed Wilcoxon rank sum test, error bars represent s.e.m.). Ctrl, control; DTP, dabrafenib/trametinib/panitumumab; DP, dabrafenib/panitumumab; TP, trametinib/panitumumab. Source data

**Extended Data Fig. 1:**
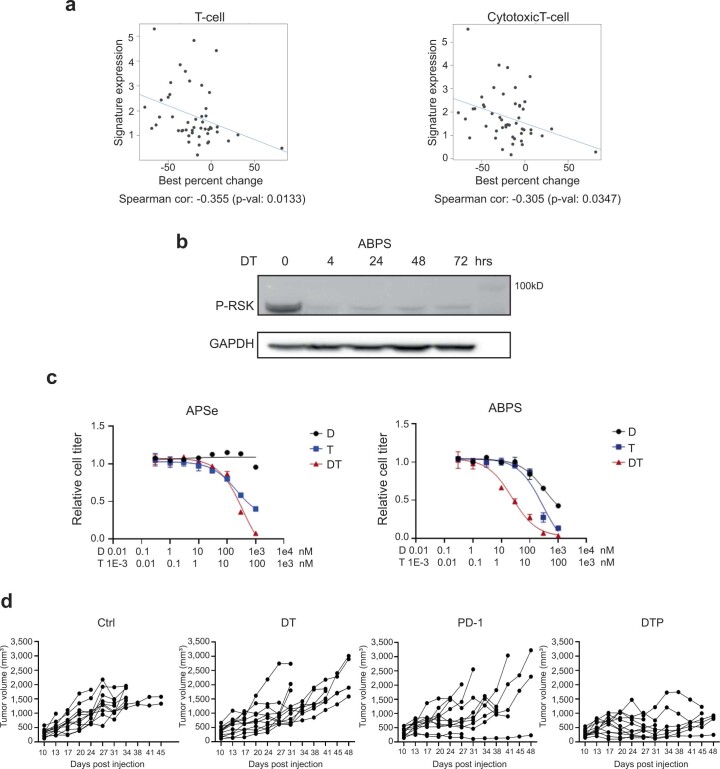
Characterization of ABPS cells. **a**, Spearman correlation (two-sided) of indicated immune signature levels at baseline and best percentage change from baseline in the size of target lesion from patients in clinical trial of dabrafenib/trametinib/panitumumab (DTP treatment arm only, n = 47). **b**, ABPS were treated with DT for 4, 24, 48, and 72 hours, and subjected to detection of phospho-RSK levels by WB (n = 3 independent biological experiments). **c**, APSe (APS cell line infected with retrovirus containing empty vector, used as control) and ABPS cells were treated with various doses of D, T, or DT for 72 hours, and subjected to cell viability assay (n = 3 independent biological experiments, data are presented as mean values +/− SEM). d, Individual growth curve of ABPS tumors across indicated treatment groups.

Our previous clinical trials with BRAF-targeted therapy combinations in BRAF^V600E^ CRC suggested potential links between BRAF pathway inhibition and the immune response^12^, including durable benefit lasting >1 year in roughly one third of patients with BRAF^V600E^ CRC also harboring MSI. To investigate this potential cooperativity, we analyzed bulk RNAseq data from 71 patients from our earlier clinical study of combined BRAF/EGFRi ± MEKi in patients with BRAF^V600E^ CRC, including 45 paired patient biopsies (pretreatment and day 15 on treatment) and 26 separate biopsies from baseline^25^. Notably, RNAseq of baseline tumor biopsies revealed significantly higher T cell signatures (indicative of increased T cell levels) in responders versus nonresponders (Fig. 1a). Baseline levels of T cell and cytotoxic T cell signatures also correlated with the best percentage change in target lesion size from baseline (Extended Data Fig. 1a). Furthermore, across all patients, increases in T cell, cytotoxic T cell and other immune signatures were noted after 15 days of treatment relative to the paired baseline biopsy, suggesting increased T cell and immune infiltration in tumors following BRAF pathway inhibition (Fig. 1b). These data support a potential interaction between BRAF/MAPK inhibition and the immune response in BRAF^V600E^ CRC.

To model this potential cooperativity, we generated a syngeneic BRAF^V600E^ CRC mouse tumor model from C57BL/6 colon organoids with knockout of Adenomatous polyposis coli (APC), TP53 and SMAD4 and expressing BRAF^V600E^ (APC, BRAF^V600E^, TP53, SMAD4 (ABPS) cells). Inhibition of MAPK signaling and sensitivity to combined BRAF/MEK inhibition with DT in ABPS cells were confirmed in vitro (Extended Data Fig. 1b,c). To assess immune effects of BRAF/MEK inhibition and the potential cooperativity with ICB, we implanted ABPS tumor cells subcutaneously into immune-competent C57BL/6 mice and treated them with vehicle, DT, anti-PD-1 antibody, or the combination. Interestingly, while BRAF/MEKi or anti-PD-1 antibody alone had minimal effect on tumor volume compared with vehicle, combined BRAF/MEK/PD-1 inhibition produced a more substantial and sustained reduction in tumor growth (Fig. 1c and Extended Data Fig. 1d). Moreover, in tumors harvested after 10 days of treatment, we detected a significant increase in the percentage of CD3^+^CD8^+^ T cells in tumors treated with BRAF/MEKi compared with vehicle control (Fig. 1d,e), suggesting that BRAF pathway inhibition alone can lead to increased T cell infiltration, similar to our observations in patient biopsies (Fig. 1b). Notably, treatment with PD-1 antibody alone did not lead to a significant increase in CD3^+^CD8^+^ T cells, although a significant increase was observed with combined BRAF/MEK/PD-1 inhibition (Fig. 1d,e). Collectively, these preclinical and translational data suggest that BRAF pathway inhibition may augment the immune response in BRAF^V600E^ CRC.

### Clinical efficacy

Based on these data, we initiated the first clinical trial, to our knowledge, combining targeted BRAF pathway inhibition with ICB in BRAF^V600E^ CRC. Patients with BRAF^V600E^ CRC were treated with the BRAFi dabrafenib, the MEKi trametinib, and the anti-PD-1 antibody spartalizumab (PDR001). While DT is not the optimal BRAF-targeted strategy for BRAF^V600E^ CRC—producing a 7% cORR and a median PFS of 3.5 months in a previous clinical trial^11^—established dosing and safety data for this triple regimen from patients with melanoma allowed for more rapid initiation of this proof-of-concept clinical trial, and we reasoned that evidence of clinical cooperativity observed would provide rationale for future evaluation of ICB combinations with more effective BRAFi combinations, including anti-EGFR antibody combinations.

**Fig. 2:**
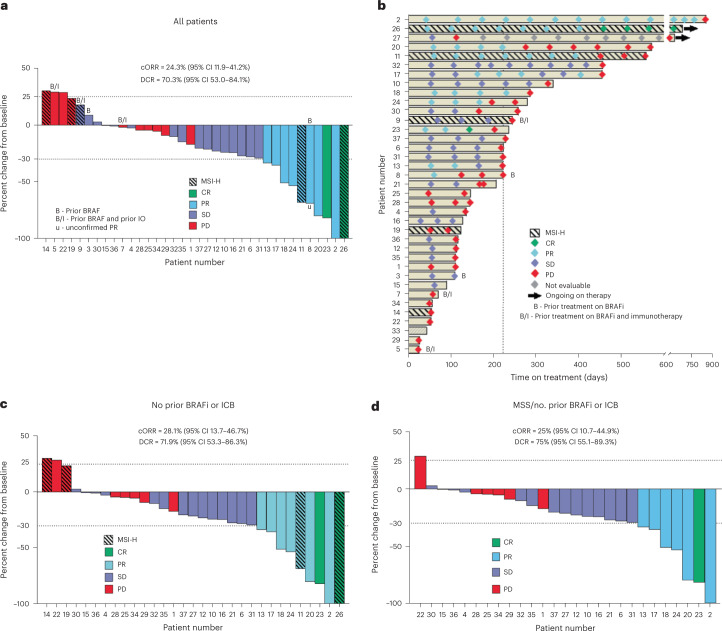
Clinical efficacy of dabrafenib, trametinib and PDR001 in patients with BRAF^V600E^ CRC. **a**, Best percentage change in the sum of the longest tumor diameters from baseline according to RECIST v.1.1 for patients in the total intention-to-treat cohort. ‘IO’, ‘MSI-H’, ‘SD’, and ‘PD’ denotes immunotherapy, microsatellite instability-high, stable disease, and progressive disease, respectively. **b**, Swimmer plot presenting the duration of treatment exposure and efficacy assessments in all patients. **c**, Best percentage change in the sum of the longest tumor diameters from baseline according to RECIST v.1.1 among patients without prior receipt of a BRAFi and/or immunotherapy in the intention-to-treat cohort. **d**, Best percentage change in the sum of the longest tumor diameters from baseline according to RECIST v.1.1 among patients without prior receipt of a BRAFi and/or immunotherapy and with microsatellite stability in the intention-to-treat cohort.

**Extended Data Fig. 2:**
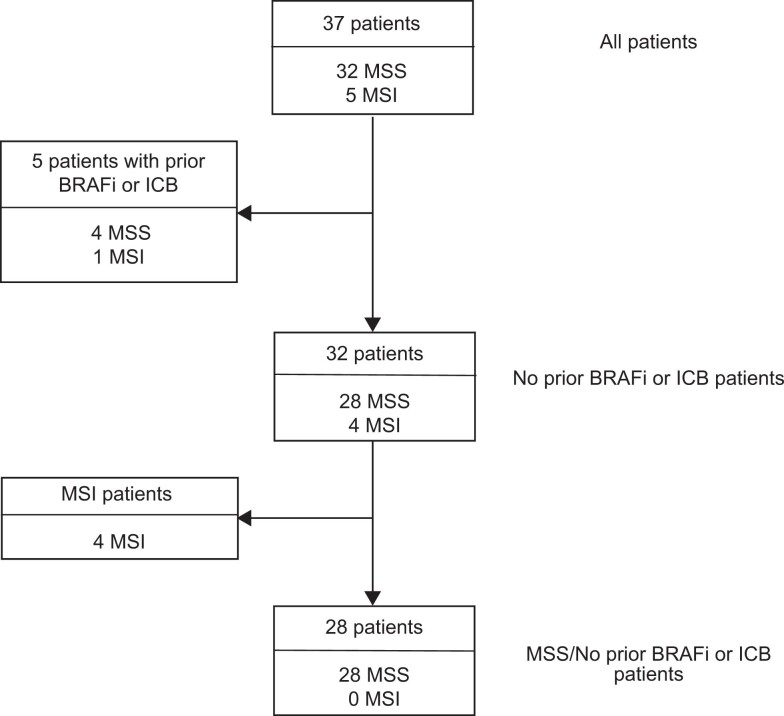
A consort style diagram of patient cohorts. **a** consort style diagram of patient cohorts.

**Extended Data Fig. 3:**
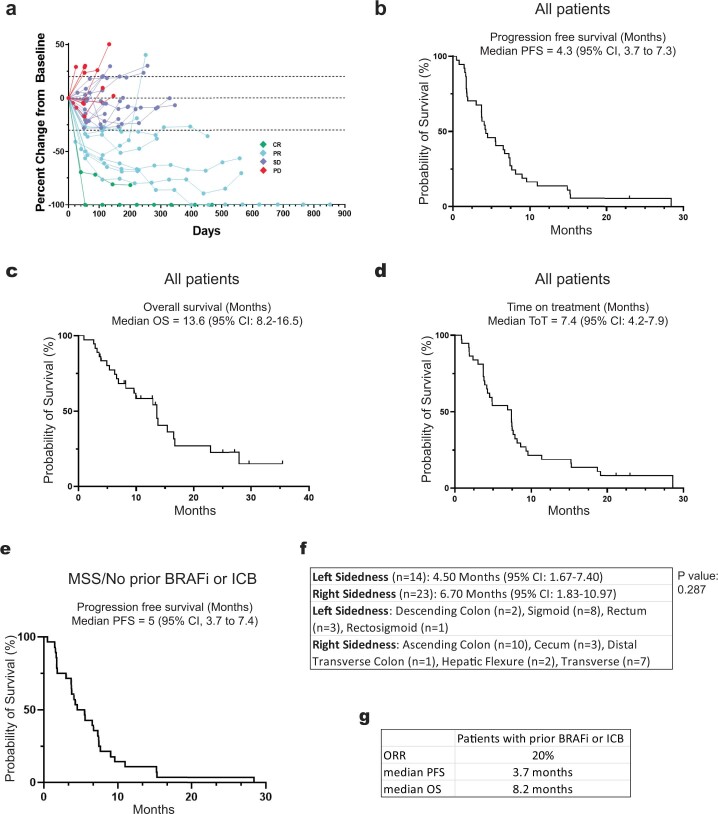
Patient survival outcomes. **a**, Best percent change from baseline over time per RECIST v1.1 criteria for patients in the total intention-to-treat cohort. **b**, Kaplan–Meier estimates of progression-free survival assessed according to RECIST v1.1 for patients in the total intention-to-treat cohort. **c**, Kaplan–Meier estimates of overall survival assessed according to RECIST v1.1 for patients in the total intention-to-treat cohort. **d**, Kaplan–Meier estimates of time on treatment assessed according to RECIST v1.1 for patients in the total intention-to-treat cohort. **e**, Kaplan-Meier estimates of progression-free survival assessed according to RECIST v1.1 for patients without prior receipt of a BRAF inhibitor and/or immunotherapy, and with microsatellite stability in the intention-to-treat cohort. **f**, difference in PFS by sidedness for patients in the total intention-to-treat cohort (Cox regression test). **g**, ORR, PFS, and OS information of patients with prior BRAFi or ICB.

As of the data cutoff, 37 of a planned 40 patients with BRAF^V600E^ CRC have been enrolled. All 32 slots for patients who are MSS accrued as well as 5 of 8 slots reserved for patients with MSI. Five patients had previous therapy with either BRAFis and/or immune checkpoint inhibitors (Extended Data Fig. 2). Median age was 63 (range 35–87), 20 (54.1%) were women (Extended Data Table 1), and median follow-up was 995 days (range 245–1,324). Overall, the regimen was well tolerated, with rash, fever and diarrhea as the most common adverse events (AEs) (Extended Data Table 2). The primary end point was ORR, and secondary end points were PFS, disease control rate (DCR), duration of response and OS. Among all 37 patients, 9 achieved a confirmed response (cORR of 24.3%; 95% confidence interval (CI) 11.9–41.2%), with 1 additional patient achieving an unconfirmed response, and the DCR was 70.3% (95% CI 53–84.1%) (Fig. 2a and Extended Data Fig. 3a), which compares favorably with the historical 7% cORR (95% CI 1.5–19.1%) of dabrafenib plus trametinib alone in BRAF^V600E^ CRC. Two patients achieved a CR. Median PFS was 4.3 months (95% CI 3.7–7.3 months) (Extended Data Fig. 3b). Median OS was 13.6 months (95% CI 8.2–16.5 months) (Extended Data Fig. 3c). Median duration of time on treatment was 7.4 months (95% CI 4.2–7.9 months) (Fig. 2b and Extended Data Fig. 3d). Among the 32 patients without previous BRAF-directed therapy or ICB, cORR was 28.1% and DCR was 71.9% (Fig. 2c). ORR, PFS and OS of patients with previous BRAF-directed therapy or ICB are shown in Extended Data Fig. 3g.

Since patients with MSI CRC may respond to ICB alone, the critical focus of this study was the 28 patients without prior BRAF-directed therapy or ICB who were also MSS and would, therefore, be predicted to have negligible response rates to ICB alone. In these patients with MSS BRAF^V600E^ CRC, cORR was 25% (95% CI 10.7–44.9%) and DCR was 75% (95% CI 55.1–89.3%) (Fig. 2d), again comparing favorably with historical controls. Median PFS was 5 months (95% CI 3.7–7.4 months), with five patients (18%) remaining on therapy for over a year (Extended Data Fig. 3e). This is in stark contrast to the earlier study of DT alone, which demonstrated a median PFS of only 3.5 months with no patients who were MSS staying on therapy greater than 1 year. One patient achieved a partial response (−100% by Response Evaluation Criteria in Solid Tumors (RECISTS) v.1.1) persisting for 2.5 years.

**Extended Data Fig. 4:**
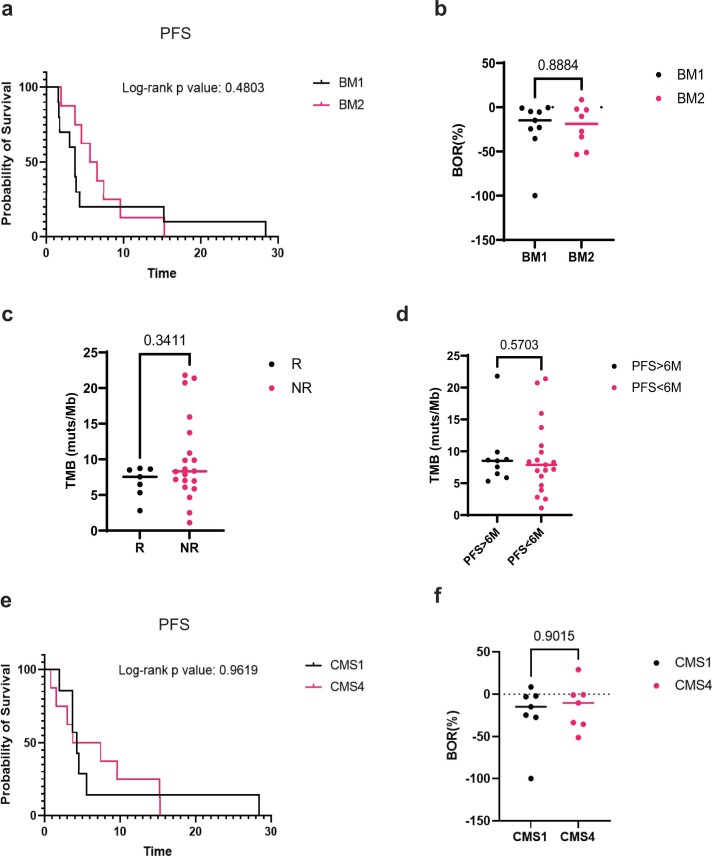
Association of baseline tumor characteristics with patient outcomes. **a** and **b**, Association of BM1/BM2 signatures with PFS (**a**) and best overall response (BOR) (**b**) in MSS patients (BM1, n = 9; BM2, n = 8; two-tailed Wilcoxon rank sum test). c and d, Association of tumor mutational burden (TMB) at baseline with response (R, n = 7; NR, n = 20; two-tailed Wilcoxon rank sum test) (**c**) and PFS (PFS > 6M, n = 9; PFS < 6M, n = 19) (**d**) in MSS patients. e and f, Association of CMS at baseline with PFS (**e**) and (**f**) in MSS patients (CMS1, n = 7; CMS4, n = 7; two-tailed Wilcoxon rank sum test).

Analysis of baseline biopsies revealed that tumor mutational burden, BM1/BM2 transcriptional subtypes and consensus molecular subtypes (CMSs)—which were previously reported to affect prognosis and response to therapy^25–27^—did not correlate with clinical outcome (Extended Data Fig. 4 and Supplementary Table 1). Furthermore, no differences in efficacy were observed based on left versus right sidedness of the primary tumor (Extended Data Fig. 3f). Overall, these data suggest promising clinical efficacy and evidence of cooperativity between BRAF/MAPK pathway inhibition and ICB.

### Tumor-intrinsic immune response and MAPK inhibition

**Fig. 3:**
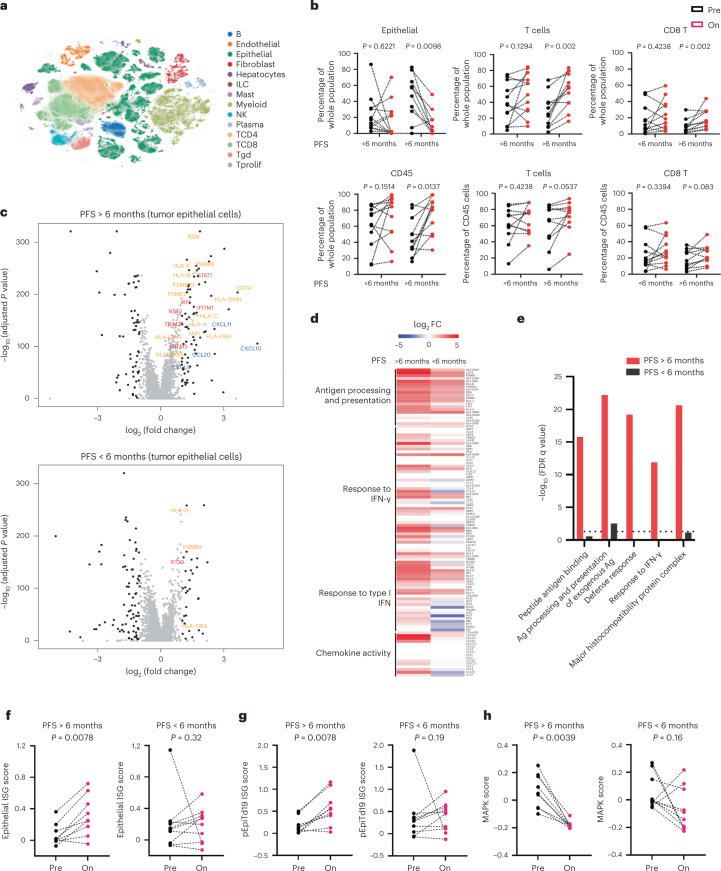
Greater tumor cell-intrinsic immune program induction and MAPK pathway inhibition in patients with better outcome. **a**, *t*-distributed stochastic neighbor embedding plot of 419,551 cells color coded for the indicated cell type. ILC, innate lymphoid cell; NK, natural killer cell; Tgd, gamma-delta T cell; Tprolif, proliferating T cell. **b**, Percentage of indicated cell types (on- versus pretreatment biopsies) in patients with PFS > 6 months (*n* = 11) and patients with PFS < 6 months (*n* = 12; two-tailed Wilcoxon rank sum tests). **c**, Volcano plots showing upregulated and downregulated DEGs (on treatment versus pretreatment) in tumor epithelial cells of patients with PFS > 6 months and PFS < 6 months. Black dots on the volcano plots indicate adjusted *P* < 0.05 (two-tailed Wilcoxon rank sum test) and log_2_FC ≥ 1. Significant DEGs involved in antigen processing and presentation (gold), the IFN pathway (red) and chemokine activity (blue) are labeled. **d**, log_2_FC (on treatment versus pretreatment) of expression of ISGs involved in indicated immune pathways in tumor epithelial cells of patients with PFS > 6 months and PFS < 6 months. **e**, Enriched immune-related Gene Ontology terms in tumor epithelial cells of patients with PFS > 6 months and PFS < 6 months. Dotted line indicates false discovery rate (FDR) = 0.05. ‘Ag’ denotes antigen. **f**–**h**, Changes of epithelial ISG score (on treatment versus pretreatment) (**f**), pEpiTd19 ISG score (on treatment versus pretreatment) (**g**) and MAPK score (on treatment versus pretreatment) (**h**) in tumor epithelial cells of patients with PFS > 6 months (*n* = 9) and PFS < 6 months (*n* = 10; Wilcoxon signed rank test).

To understand the potential interaction of BRAF/MAPK pathway inhibition and the tumor immune response, all patients underwent paired pretreatment and day 15 on-treatment biopsies of the same tumor lesion. Fresh tumor biopsies were analyzed by scRNAseq with evaluable data obtained from both paired biopsies in 23 patients. A total of 419,551 single cells passed quality control (QC) across all specimens and stromal, immune and tumor epithelial cell populations (Fig. 3a). Comparing changes in the abundance of individual cell populations in pre- versus on-treatment biopsies, we observed a significant decrease of tumor epithelial cells as well as an increase of CD45^+^ immune cells (defined by cell-type clustering), T cells and CD8^+^ T cells after treatment in patients with PFS > 6 months (*n* = 11) but not in patients with PFS < 6 months (*n* = 12) (Fig. 3b).

**Extended Data Fig. 5:**
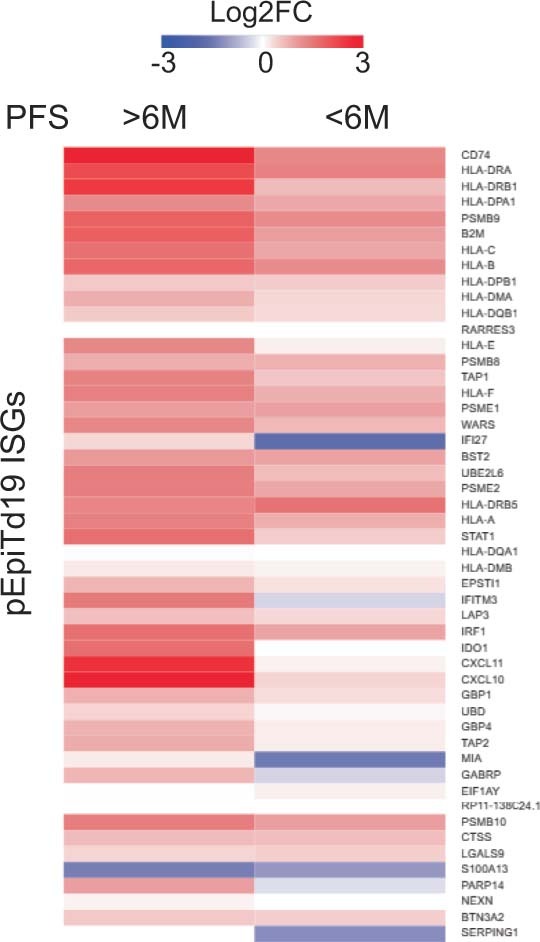
Expression of ISGs in patient tumor biopsies. Log2 fold change (on- versus pre-treatment) of expression of top 50 genes from pEpiTd19 ISG program in tumor epithelial cells of patients with PFS > 6 months and < 6 months.

To understand the effects of BRAF/MAPK inhibition specifically in tumor cells, we characterized gene expression changes after treatment in the tumor epithelial compartment to identify potential tumor cell-intrinsic mechanisms underlying patient response. Evaluating differentially expressed genes (DEGs) in tumor cells at day 15 versus pretreatment revealed striking increases in the expression of immune-related genes in patients with PFS > 6 months that were not observed in patients with PFS < 6 months, including genes involved in interferon (IFN) response (for example, *STAT1*, *IRF1*, *IFITM1*, *IFITM3*, *BST2* and *TRIM31*), antigen processing and presentation (for example, *B2M*, *CD74*, *PSMB10*, *TAP1*, *HLA-A*, *HLA-C*, *HLA-F*, *HLA-DRB1* and *HLA-DPA1*) and chemokine activity (for example, *CXCL9*, *CXCL10* and *CXCL11*) (Fig. 3c,d; all DEGs are listed in Supplementary Table 2), suggesting global upregulation of IFN-stimulated transcriptional programs and antigen processing and presentation pathways, which was also observed by gene set enrichment analyses (Fig. 3e and Supplementary Table 3). In contrast, fewer immune-related gene sets were enriched in tumor epithelial cells in patients with PFS < 6 months (Fig. 3e). DEGs in patients with PFS > 6 months that mapped to antigen processing and presentation, response to IFN-γ, response to type I IFN, and chemokine activity programs were used to create a score of epithelial interferon-stimulated genes (ISGs) (Fig. 3d and Supplementary Table 4). This score was significantly elevated on treatment in patients with PFS > 6 months but not PFS < 6 months in a patient-level analysis (in addition to the gene-level analysis shown before) (Fig. 3f). We also assessed a score for a human CRC malignant epithelial-specific ISG program we recently derived in an independent scRNAseq effort and that was associated with activated and chronically stimulated T cells^28^. Again, patients with PFS > 6 months showed significantly increased scores (pEpiTd19 ISG) at day 15, whereas patients with PFS < 6 months did not (Fig. 3g and Extended Data Fig. 5).

We next examined the degree of MAPK pathway inhibition after treatment in tumor cells using an MAPK gene expression signature score based on changes in the MAPK-regulated transcripts (*DUSP6*, *ETV4*, *ETV5* and *SPRY4*) in tumor epithelial cells. Interestingly, the MAPK score was significantly reduced after treatment in tumor epithelial cells of patients with PFS > 6 months but not in patients with PFS < 6 months (Fig. 3h). Thus, scRNAseq analysis of tumor cell-intrinsic gene expression changes following treatment revealed that patients with longer PFS showed greater induction of tumor-intrinsic immune programs as well as greater MAPK pathway inhibition.

### Enhanced immune response driven by optimized MAPK inhibition

**Extended Data Fig. 6:**
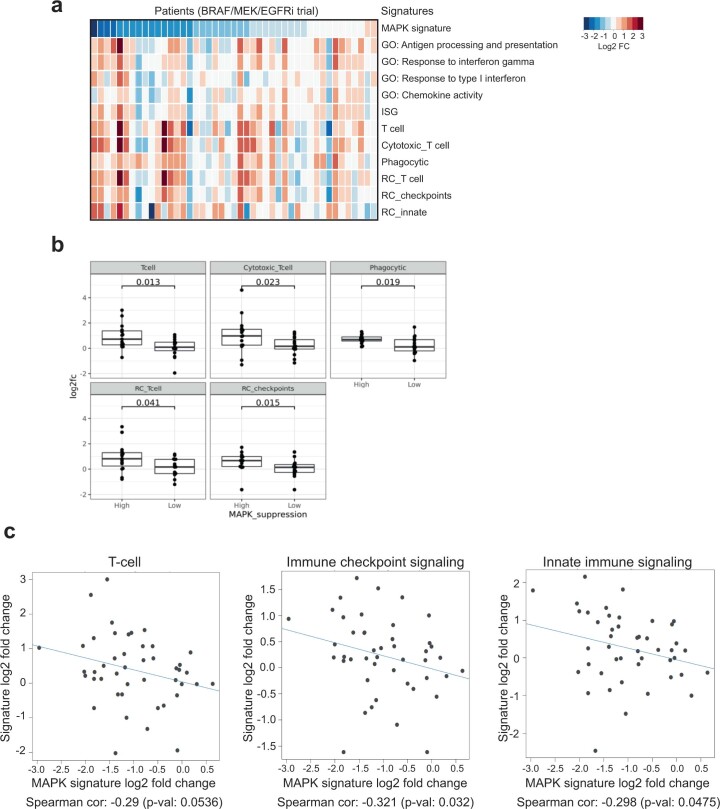
Immune signatures in patients treated with BRAF targeted therapy. **a**, Log2 fold change of immune signatures in patients ordered by the degree of MAPK pathway inhibition (left to right: strong to weak inhibition) in previous BRAF/MEK/EGFRi trial (all DTP, DP, TP treatment arms). **b**, Two-tailed Wilcoxon rank sum test of indicated immune signatures between patients with top and bottom 1/3 in MAPK suppression from BRAF/MEK/EGFRi trial (High, n = 15; Low, n = 15). The box plots show the median and Q1 and Q3 (25th and 75th percentiles) of the data; the upper and lower whiskers extend to the min and max values no further than 1.5× the interquartile range (IQR), respectively; and outliers are plotted individually. **c**, Spearman correlation (two-sided) of log2 fold change (on- versus pre-treatment) of indicated immune signature levels and log2 fold change (on- versus pre-treatment) of MAPK signature levels in BRAFV600E CRC patients from the BRAF/MEK/EGFRi trial (all treatment arms, n = 45).

We therefore hypothesized that the degree of MAPK inhibition achieved in tumor cells might be directly related to the degree of tumor-intrinsic induction of immune gene expression. Accordingly, we evaluated the effects of BRAF/MAPK pathway inhibition alone in patients with BRAF^V600E^ CRC treated on previous studies with BRAF targeted therapy only. We analyzed RNAseq data from 45 paired pretreatment and on-treatment (day 15) biopsies from patients with BRAF^V600E^ CRC from a previous clinical trial of BRAF/MEK/EGFR inhibition^12,25^. Notably, the induction of several immune signatures at day 15 correlated with the degree of MAPK pathway inhibition (Extended Data Fig. 6a). These included T cell and cytotoxic signatures, immune checkpoint signaling and phagocytic signatures (Extended Data Fig. 6b). Furthermore, we also found correlation between the degree of MAPK inhibition and immune signature induction in on-treatment versus pretreatment biopsies, including T cell, immune checkpoint and innate immune response signatures (Extended Data Fig. 6c). The fact that a greater increase in immune signatures was observed in patients with BRAF^V600E^ CRC with a greater degree of MAPK pathway inhibition supports the hypothesis that the degree of MAPK inhibition in tumor cells may drive immune gene induction and aspects of the tumor immune response.

**Fig. 4:**
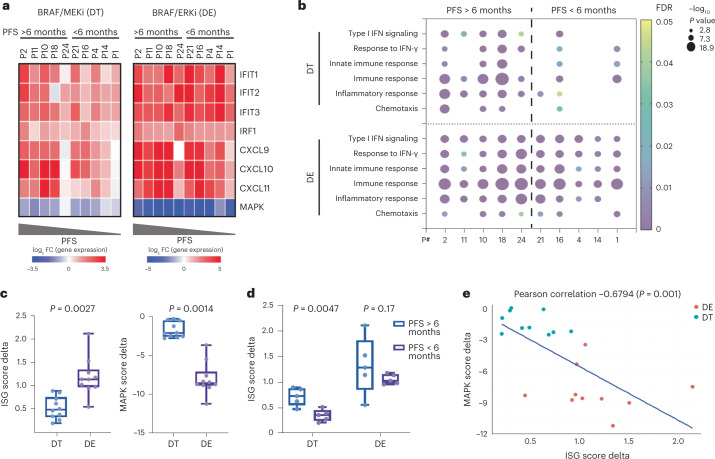
Enhanced tumor cell-intrinsic immune program induction is driven by optimized MAPK inhibition. **a**, The log_2_FC of gene expression (measured by qPCR) of indicated ISGs and the average of log_2_FC of MAPK-regulated transcripts in patient-derived organoids treated with DT or DE for 72 h. Organoids are arranged based on the PFS data of patients (from left to right: longest to shortest PFS). **b**, GO term enrichment analysis of upregulated DEGs (log_2_FC ≥ 1, *P* < 0.05, Fisher exact test) in organoids treated with 72-h DT or DE versus control. Gene expression was measured by bulk RNAseq. Organoids are arranged based on patient PFS data. **c**, Delta of ISG scores (left) and MAPK scores (right; 72-h treatment versus control) in DT-treated (*n* = 10) and DE-treated (*n* = 10) organoids (two-tailed Wilcoxon rank sum test). **d**, Delta of ISG scores (72-h treatment versus control) in organoids derived from patients with PFS > 6 months (*n* = 5) and PFS < 6 months (*n* = 5; two-tailed Wilcoxon rank sum test). In **c** and **d**, the box plots show the median, Q1 and Q3 (25th and 75th percentiles) of the data; the upper and lower whiskers extend to the minimum and maximum values. **e**, Pearson correlation (two sided) of ISG score delta and MAPK score delta in all DT- and DE-treated organoids.

**Extended Data Fig. 7:**
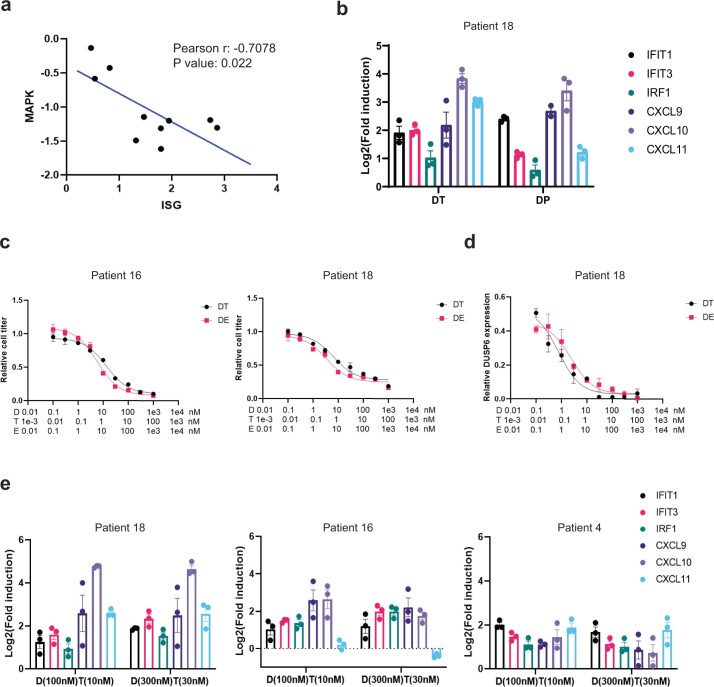
Characterization of organoids following BRAF targeted therapy. **a**, Pearson correlation (two-sided) of ISGs induction (average of log2FC of ISGs in Fig. 4a) and MAPK pathway inhibition (average of log2FC of DUSP6, ETV4, ETV5, and SPRY4) in DT-treated organoids. **b**, Log2FC of gene expression (measured by qPCR) of indicated ISGs in organoids (derived from patient 18) treated with DP for 72 hours (n = 3 independent biological experiments, data are presented as mean values +/− SEM). **c**, Indicated organoids were treated with various doses of DT or DE for 72 hours, and subjected to cell viability assay (n = 3, data are presented as mean values +/− SEM). **d**, Relative DUSP6 expression in organoid treated with various doses of DT or DE for 6 hours (n = 3, data are presented as mean values +/− SEM). e, ISGs induction in indicated organoids treated with two different doses of DT (n = 3, data are presented as mean values +/− SEM).

To evaluate the potential relationship between MAPK inhibition and immune program induction specifically within tumor cells, we utilized patient-derived organoid models successfully generated from baseline tumor biopsies from 10 patients, including 5 with PFS > 6 months and 5 with PFS < 6 months. Organoids were treated with DT and gene expression was measured by quantitative polymerase chain reaction (qPCR). Organoids derived from patients with PFS > 6 months showed greater increases in the expression of genes involved in IFN response (*IFIT1*, *IFIT2*, *IFIT3* and *IRF1*) and chemokine activity (*CXCL9*, *CXCL10* and *CXCL11*) compared with organoids derived from patients with PFS < 6 months (Fig. 4a, left). Importantly, organoids from patients with PFS < 6 months also showed a lesser degree of MAPK pathway inhibition (calculated by the average *DUSP6*, *ETV4*, *ETV5* and *SPRY4* log_2_FC) after treatment with DT (Fig. 4a, left). The degree of ISG induction was significantly correlated with the degree of MAPK inhibition (Extended Data Fig. 7a), suggesting that insufficient MAPK pathway inhibition could potentially explain the differences in ISG upregulation. Notably, the varying degree of ISG induction and MAPK inhibition by DT in both groups of organoids (PFS > 6 months or PFS < 6 months) mirrored the differences observed from scRNAseq analysis of the tumor epithelial compartments in patients from the same groups. Similarly, global transcriptomic profiling of organoids treated with DT by RNAseq showed significant induction of more immune gene sets in organoids derived from patients with PFS > 6 months compared with organoids from patients with PFS < 6 months (Fig. 4b). Likewise, treatment of organoid models with dabrafenib and the anti-EGFR antibody panitumumab also led to a similar induction of ISGs (Extended Data Fig. 7b). Importantly, these data confirm that MAPK pathway inhibition can drive induction of immune gene expression in a tumor cell-intrinsic manner that does not depend on stimuli from the tumor immune microenvironment since organoid cultures contain tumor cells only and do not contain immune or stromal cells from the tumor microenvironment. These data also provide further correlative evidence that the degree of MAPK suppression may be directly related to the degree of immune gene induction.

To test whether more optimal and complete inhibition of the BRAF/MAPK pathway might enhance the degree of immune gene induction across all tumor models, including those from patients with PFS < 6 months, we utilized a combination of BRAFi and ERKi. Our earlier work showed that ERK inhibitors are able to achieve more complete MAPK pathway inhibition in combination with BRAFis compared with MEK inhibitors^29^. Thus, we treated the same collection of organoids with dabrafenib and the ERK inhibitor ERAS007 (DE). The concentrations of DT and DE chosen were based on inhibitory concentration (IC)90 values demonstrating equivalent efficacy to ensure an appropriate comparison (Extended Data Fig. 7c,d). Interestingly, when treated with DE, all organoids showed robust MAPK inhibition, regardless of the clinical outcome of the patient from whom the organoid was derived (Fig. 4a, right). BRAF/ERK inhibition also strikingly induced the expression of immune response genes to a comparable extent in all organoids (Fig. 4a, right), suggesting that higher ISG induction could be achieved by more complete MAPK inhibition, especially in organoids with lesser response to BRAF/MEKi. Notably, even with a higher concentration of DT, we still did not observe stronger induction of immune programs in organoids, including in representative models from PFS < 6 months (Extended Data Fig. 7e). Additionally, BRAF/ERK inhibitor (ERKi) treatment universally induced strong enrichment of immune-related Gene Onotology (GO) terms in organoids derived from patients with PFS > 6 months and PFS < 6 months (Fig. 4b), in contrast to observations with BRAF/MEKi.

Overall, BRAF/ERKi induced a significantly greater degree of ISGs upregulation and MAPK inhibition than BRAF/MEKi in all organoids, as reflected by a greater ISG score and MAPK score change with treatment versus control (Fig. 4c). Furthermore, whereas BRAF/MEKi led to a significantly greater ISG score increase in organoids derived from patients with PFS > 6 months versus patients with PFS < 6 months, BRAF/ERKi led to a comparable degree of ISG score induction in both groups of organoids that was not significantly different. Notably, the levels of ISG score induction in both groups following BRAF/ERKi were equal to or greater than the degree of induction seen with BRAF/MEKi in patients with PFS > 6 months (Fig. 4d). ISG score delta was strongly and significantly anticorrelated with MAPK score delta (Fig. 4e). Together, these data demonstrate that (1) effective MAPK inhibition alone can induce tumor cell-intrinsic immune gene expression and that (2) improving the degree of MAPK inhibition achieved can increase the degree of tumor-intrinsic immune gene expression in all tumor models to the same levels observed in patients with PFS > 6 months with BRAF/MEKi.

Overall, these data provide evidence for a tumor cell-intrinsic role of BRAF/MAPK pathway inhibition in promoting tumor immune response. Given the relationship between the degree of MAPK inhibition and induction of immune program gene expression in tumor cells, these findings also raise the possibility that improved MAPK inhibition through a more optimal BRAF-targeted combination could drive greater immune cooperativity and greater clinical efficacy in combination with ICB.

## Discussion

We present preclinical, clinical and translational evidence of cooperativity between BRAF/MAPK pathway inhibition and the immune response in BRAF^V600E^ CRC. Importantly, even in patients with MSS BRAF^V600E^ CRC, in whom a negligible response rate to ICB is expected, the combination of PD-1, BRAF and MEK inhibition yielded more than a threefold increase in cORR (25%, 95% CI 10.7–44.9%) relative to historical controls of combined BRAF/MEK alone (7%, 95% CI 1.5–19.1%) in patients without prior BRAFi. This also compares favorably with the 20% cORR of encorafenib plus cetuximab, the current Food and Drug Administration-approved standard for BRAF^V600E^ CRC. We also observed evidence of increased durability with a median PFS of 5 months (versus 3.5 months with BRAF/MEK alone), with 57% of patients remaining on treatment for >6 months and 18% for >1 year.

The primary focus of the study was efficacy in the MSS BRAF^V600E^ CRC population since these patients have a near-zero response to ICB and thus, the greatest clinical need. However, eight slots were reserved for patients with MSI BRAF^V600E^ in an effort to assess whether combined BRAF pathway targeting might enhance the basal responsiveness of MSI tumors to ICB. While durable responses were observed in two of five patients with MSI enrolled, this 40% ORR does not suggest clear evidence of enhanced clinical benefit, and enrollment to remaining MSI slots is ongoing.

In what we believe is one of the first clinical trials to incorporate systematic scRNAseq analysis of paired pre- and on-treatment tumor biopsies from all patients, we identified a potential mechanism underlying the cooperativity observed between BRAF/MAPK inhibition and immune response. We find evidence of tumor cell-intrinsic induction of key immune programs (types I and II IFN response, antigen presenting genes and T cell recruiting chemokines) triggered by MAPK pathway inhibition. These gene expression changes are similar to a gene expression program recently discovered in malignant cells from immunologically active MSI CRC and associated with activated and chronically stimulated T cells^28^. Interestingly, induction of T cell recruiting chemokines, such as CXCL9/10/11 and certain ISGs, has also been observed in preclinical CRC mouse models following KRAS^G12C^ inhibition^30^, suggesting that this tumor cell-intrinsic transcriptional response may be a key mechanism more broadly linked to inhibition of the RAS–MAPK pathway. Our scRNAseq data revealed that upregulation of immune programs in tumor cells was significantly greater in patients with prolonged PFS, suggesting that this effect may be a critical mechanism mediating therapeutic benefit.

By performing parallel studies with matched patient-derived organoids generated from the same patients, we confirmed that induction of immune genes by BRAF/MAPK inhibition is tumor cell intrinsic and does not depend on cells in the tumor microenvironment. Indeed, induction of these immune programs was observed even in organoid cultures, which consist of pure tumor cell populations. These data provide important mechanistic insight, as the potential cooperativity of MAPK pathway inhibition and the immune response has previously been hypothesized to involve a direct effect of MAPK inhibition on cells in the tumor microenvironment^18^. One potential limitation of the current study is that it focused on the tumor cell compartment. It is still possible that MAPK inhibition within the tumor microenvironment may affect the tumor immune response, and a detailed assessment of changes in the abundance and gene expression profiles of other cell types will be undertaken through analysis of our scRNAseq data, coupled with parallel methods, in a future study. However, our data provide evidence for a robust tumor cell-intrinsic mechanism and support that BRAF/MAPK pathway activation promotes immune suppressive signals within tumor cells that can be reversed with targeted pathway inhibition. More detailed mechanistic experiments in future studies may further delineate the importance and relative contribution of this tumor cell-intrinsic mechanism to the tumor immune response.

Notably, organoids derived from patients with PFS > 6 months also retained the pattern of increased induction of immune programs upon BRAF/MEK treatment relative to organoids from patients with PFS < 6 months, as observed in our scRNAseq dataset. This observation allowed us to use these matched organoids as representative models to probe the mechanisms underlying tumor cell-intrinsic immune program induction. Interestingly, our scRNAseq data suggested that patients with PFS < 6 months failed to achieve robust MAPK inhibition in tumor cells with DT compared with patients with PFS > 6 months. This same pattern was observed in matched patient-derived organoid models, allowing us to test the hypothesis that failure to induce immune programs was a direct consequence of inadequate BRAF/MAPK inhibition and that more optimal MAPK inhibition may lead to improved induction of key immune genes, even in tumor cells from patients who did not benefit from therapy. As noted above, a limitation of the study was the use of a BRAF/MEK inhibitor combination for this proof-of-concept study, which was based on the availability of established dosing and safety data for this regimen from patients with melanoma at the time that this study was initiated; this preceded the results of the BEACON study that demonstrated the efficacy of encorafenib and cetuximab in this population. Notably, the BEACON study also showed that while the addition of an MEK inhibitor increased the overall response rate relative to the BRAF/EGFR doublet (27% versus 20%), it did not improve survival. Thus, BRAF/MEK is likely not the optimal BRAF-targeting core in this population, and these data suggest that more effective BRAF-targeting combinations could further augment the cooperativity with ICB. Remarkably, using a combination of BRAF and ERK inhibitors that led to more robust BRAF/MAPK inhibition across all organoids, we found that immune program induction could be achieved in all models, regardless of PFS. This finding has important and immediate clinical implications, suggesting that combining ICB with a more effective BRAF/MAPK-targeting core, such as BRAF/EGFR or BRAF/ERK, may further enhance the immune cooperativity and clinical benefit observed and that such strategies warrant further exploration in future clinical trials. This potential for enhanced clinical activity from targeting the BRAF pathway in combination with ICB is further supported by early data from an ongoing clinical trial adding the anti-PD-1 antibody nivolumab to the current standard of care of encorafenib plus cetuximab, yielding a 50% ORR in the first 21 patients compared with an expected 20% ORR for encorafenib plus cetuximab alone^31^.

Finally, it will be important to determine whether this potential mechanism of immune cooperativity is limited to BRAF^V600E^ CRC or whether it would apply to other tumor types and to other agents targeting the MAPK pathway. For example, combinations of ICB (PD-1 or programmed death-ligand (PD-L)1) with BRAFi/MEKi in BRAF^V600^ melanoma have also suggested improved benefit, although each component of this combination exhibits greater activity alone in melanoma than in CRC. Notably, a large phase III study of the MEK inhibitor cobimetinib and the anti-PD-L1 antibody atezolizumab in CRC failed to achieve its primary end point, which has cast doubt on whether MAPK pathway inhibition in general has the potential to enhance the immune response. However, this trial was performed in all CRC genotypes (including CRC without MAPK-activating mutations), and data suggest that MEK inhibitors alone fail to maintain MAPK inhibition in CRC due to adaptive feedback^32^. Preclinical data support that induction of similar immune gene programs as observed in our study can be found in mouse models of KRAS^G12C^ CRC with KRAS^G12C^ inhibitor treatment^30,33^. Therefore, it is possible that the mechanism of immune cooperativity we propose in our study may be more broadly applicable to other effective RAS/BRAF/MAPK pathway inhibitors, and further studies to evaluate this possibility will be important.

## Methods

### Study design

This research study is a phase II clinical trial to test the safety and effectiveness of DT in combination with the anti-PD-1 antibody PDR001 in patients with metastatic CRC characterized by the BRAF V600E mutation (NCT03668431). Patients at the Massachusetts General Hospital Cancer Center and the Dana-Farber Cancer Institute were treated with spartalizumab (PDR001) 400 mg intravenous q28d (every 28 days), dabrafenib 150 mg oral administration twice a day for 28 consecutive days, and trametinib 2 mg oral administration daily for 28 consecutive days (dose safety was established in patients with melanoma). The study was conducted in accordance with the Guidelines for Good Clinical Practice and the ethical principles described in the Declaration of Helsinki, and it was approved by the local institutional review board.

### Patients

Eligible patients must have histologically or cytologically confirmed metastatic CRC, have a documented BRAF V600E mutation by a CLIA-certified laboratory test and be wild type for KRAS and NRAS. Patients were required to be aged ≥18 years, have measurable disease according to RECIST v.1.1, have an Eastern Cooperative Oncology Group performance status of less than or equal to two and have adequate baseline organ function (as determined by laboratory parameters). The first patient was enrolled on 15 October 2018. The trial was amended after the first nine patients to exclude patients with prior BRAF or MEK inhibitor or immunotherapy, and this amendment was Institutional Review Board approved. Enrollment of the remaining slots reserved for patients with MSI on the trial is still ongoing at this time. Key exclusion criteria included chemotherapy or radiotherapy within 4 weeks prior to entering the study and any serious or unstable preexisting medical condition. All patients provided written informed consent before the study.

### Efficacy assessments

Patients received study therapy until disease progression, unacceptable toxicity, death or discontinuation for any other reason. Safety was monitored throughout the study for all patients across cohorts via physical examinations, laboratory evaluations, vital sign and weight measurements, performance status evaluations, ocular and dermatologic examinations, concomitant medication monitoring, electrocardiograms, echocardiograms and AE monitoring (characterized and graded per Common Terminology Criteria for Adverse Events v.4.0). AEs were recorded using the standard Medical Dictionary for Regulatory Activities coding. Dose interruptions, reductions and discontinuations for all of the study drugs were monitored.

The primary end point was ORR; secondary end points were PFS, disease control rate, duration of response and OS, and the exploratory end point was scRNAseq analysis. Antitumor efficacy was assessed by CT or MRI at baseline and then, every 8 weeks until progression or death. Response determination was based on RECIST v.1.1 by the Dana-Farber/Harvard Cancer Center Tumor Metrics Core. For the subset of patients who showed a confirmed CR or PR, duration of response was defined as the time in weeks from the first documented evidence of CR or PR (the first response prior to confirmation) until the time of documented disease progression or death due to any cause, whichever was first. PFS was defined as the time in weeks between the first dose and the date of disease progression or death due to any cause. Finally, OS was defined as the time in weeks from the first dose of study drug until death due to any cause. PFS and time on treatment were summarized with Kaplan–Meier methodology using medians and 95% CIs (estimated using the Brookmeyer–Crowley method). Time on treatment was defined as the time until final treatment discontinuation. Fresh tumor biopsies were collected before dose (day 1) for scRNAseq analysis and patient-derived organoids generation as well as after dose (day 15) for scRNAseq analysis. The same tumor lesion was biopsied at baseline and at day 15. Formalin-fixed paraffin-embedded (FFPE) and flash-frozen tumor samples were collected at day 1 for genomic and molecular analyses.

### Whole-exome sequencing and TMB analysis

For each biopsy, we called somatic point mutations against the patient’s matched blood normal control sample using MuTect1^34^ for single base substitutions and Strelka2^35^ for indels. Mutations were filtered for sequencing artifacts using the Getz Lab whole-exome analysis pipeline. We then calculated tumor mutation burden (TMB) in terms of mutation density by dividing the total number of mutations called for each biopsy by the total captured exonic territory from the TWIST Biosciences bait set.

### BM1 and BM2 signatures and CMS analysis

We aligned raw RNAseq reads using STAR^36^ two-pass transcriptome/genome alignment and then quantified per-transcript counts using RSEM^37^. We then performed Bayesian nonnegative matrix factorization (BNMF)^38^ on the matrix of transcript counts for each sample. This expresses the vector of each sample’s transcript counts as a linear combination of vectors corresponding to transcriptional signatures common across samples, with the overall number of signatures automatically determined by the Bayesian model hierarchy. We identified one BNMF signature whose genes closely matched the gene set defining the BM1 signature^26^ and another BNMF signature whose genes closely matched the gene set defining the BM2 signature. We found that the sample loadings for these two signatures were nearly mutually exclusive among samples (that is, a sample with a high BM1 loading almost always had a negligible BM2 loading and vice versa), allowing us to classify the majority of samples by their BM1/BM2 status. The log_2_ TPM counts from RSEM were passed directly to the CMS classifier using a centroid-based predictor method, which classifies samples based on their similarity to expression clusters derived from the ground truth dataset.

### Generation of ABPS and APSe cell line

Organoids were established from colon tissue of C57BL/6 mice harboring a conditional floxed *Trp53* allele, infected with Cre-expressing adenovirus for *Trp53* deletion, subjected to CRISPR–Cas9 knockout of *Apc* and *Smad4* and dissociated to generate the APC, TP53, SMAD4 (APS) cell line. APS cells were cultured in Dulbecco’s Modified Eagle Medium (DMEM)/F12 media supplemented with 10% fetal bovine serum (FBS) and 2 mM GlutaMAX (Thermo Fisher Scientific). The BRAF V600E sequence was cloned in the pMXs-Puro Retroviral Expression Vector (Cell Biolabs). Retrovirus containing the BRAF V600E sequence or empty pMXs-Puro vector was produced in HEK293 cells with packaging vector pCL-10A1 and concentrated with Retro-Concentin Retro Concentration Reagent (System Biosciences). APS cells were cultured to 50% confluence and then infected with retrovirus using 5 μg ml^−1^ polybrene. After 48 h of infection, 2 μg ml^−1^ puromycin was added to the media to select stable ABPS (contains BRAF V600E sequence) and APC, TP53, SMAD4, empty vector (APSe) cells (used as control).

### Animal studies

ABPS cells were resuspended in PBS and Corning Matrigel in a 1:1 ratio and then injected (2.5 × 10^5^ cells per injection) into the flanks of 12-week-old male C57BL/6 mice (Charles River Laboratories). When the tumor size reached 150–200 mm^2^, mice were randomized into four groups and treated with (1) vehicle control (0.5% hydroxypropyl-methylcellulose + 0.2% Tween 80, oral gavage) and immunoglobulin G isotype control (BioXcell, intraperitoneal injection); (2) dabrafenib (30 mg kg^−1^ daily, oral gavage) and trametinib (1 mg kg^−1^ daily, oral gavage); (3) anti-PD-1 (10 mg kg^−1^ twice per week, intraperitoneal injection); or (4) dabrafenib, trametinib and anti-PD-1. The mice were treated for up to 60 days, and tumor volumes were assessed twice per week and determined according to the formula length (*L*) × width (*W*)^2^ × π/6. Animal studies and procedures were performed in accordance with the institutional guidelines of Massachusetts General Hospital, and all experiments were conducted according to institutional animal care and use committee-approved protocols. The housing conditions for mice are 20 °C to 26 °C, a 12-h light/12-h dark cycle, and 40–60% humidity.

### Immunofluorescence staining

Mouse tumors were collected and fixed in 10% formalin, embedded and sectioned (5 μm). Tumor tissue FFPE slides were then deparaffinized and rehydrated. For antigen retrieval, slides were maintained in antigen unmasking solution (Vector Laboratories; H-3300) at a subboiling temperature for 20 min using a microwave. Slides were washed three times for 5 min each in PBS supplemented with 0.1% Tween 20 and then were blocked with 5% BSA (Thermo Fisher Scientific) and 5% goat serum (Sigma-Aldrich) for 1 h at room temperature. The slides were incubated with anti-CD3 (Abcam; ab11089; 1:800 dilution) and anti-CD8 (Cell Signaling; 98941 S; 1:400 dilution) primary antibodies overnight at 4 °C. The next day, slides were washed as above and incubated with conjugated secondary antibodies (Thermo Fisher Scientific; A-11036 and A-11006) for 1 h at room temperature. The slides were then washed, stained for DAPI (Invitrogen) and mounted with SlowFade Diamond Antifade Mountant (Invitrogen). Once finished, the slides were scanned using a ZEISS Axio Scan slide scanner and analyzed using Halo software (Indica Labs). Image annotations were performed in a blinded manner. Cells stained with an intensity exceeding the settings threshold were counted as positive. The settings were set to include the full range of staining intensity (weak to strong). Halo counted the CD3^+^CD8^+^ cells in stromal and tumor, and data were collected as the number of positive cells divided by the total DAPI + cells in the tumor area.

### Organoid generation, culture and treatment

Patient-derived organoid generation was attempted from baseline biopsies of all patients. A total of 10 colorectal tumor organoid lines were successfully generated from tumor baseline biopsies of 10 patients enrolled in the BRAF/MEK/PD-1 inhibition trial (patients 1, 2, 4, 10, 11, 14, 16, 18, 21 and 24). Tumor biopsies were transported in ice-cold RPMI with 10% human serum and transferred into a petri dish on ice before processing. Tumor biopsies were minced and subjected to enzymatic dissociation in 4.75 ml minimum essential medium for suspension cultures (Gibco) supplemented with 250 µl Liberase for 45 min at 37 °C using a heater-shaker. Following the dissociation, tumor biopsies were centrifuged at 300*g* for 5 min, seeded into Matrigel in a prewarmed 24-well plate and cultured with 500 µl of basal growth media. For passaging, organoids were mechanically pipetted out of Matrigel using Corning Cell Recovery Solution (Corning), followed by a 1-h incubation at 4 °C. Organoid fragments were then centrifuged and subjected to enzymatic dissociation in Tryple E (Gibco) for 5 min at 37 °C. A 20-gauge needle was used to further disrupt the organoids mechanically. DMEM/F12 media supplemented with 10% FBS was added to the conical tube to stop the enzymatic reaction. Dissociated organoids were collected by centrifugation and seeded in Matrigel as above; 10 µM Rko-Kinase inhibitor was added to the basal growth media for organoid passaging. For drug treatment, dissociated organoids were resuspended in basal growth media supplemented with 2% Matrigel and plated in a 24-well plate coated with 250 µl Matrigel. The next day, organoids were treated with dabrafenib (100 nM) + trametinib (10 nM), dabrafenib (100 nM) + ERAS007 (100 nM), or dabrafenib (100 nM) + panitumumab (3 µg ml^−1^; McKesson) for 72 h. The drugs were refreshed every 24 h. After the treatment, organoids were collected and subjected to RNA extraction. For cell viability experiments, dissociated organoids supplemented with 2% Matrigel were plated in a 96-well plate coated with 70 µl Matrigel; 48 h later, organoids were treated with various doses of dabrafenib + trametinib or dabrafenib + ERAS007 for 72 h and subjected to cell viability measurement using CellTiter-Glo 3D (Promega).

### Organoid basal growth media

Organoid basal growth media consist of 30% DMEM/F12 media supplemented with 20% FBS, 50% WNT3A conditioned media, 20% R-spondin conditioned media, 1× B27 (Life Technologies; 17504-044), 1; N2 (Life Technologies; 17502-048), 10 mM nicotinamide (Sigma; N0636), 1.25 mM *N*-acetyl-L-cysteine (Sigma; A9165), 100 µg ml^−1^ Primocin (InvivoGen; ant-pm,2), 0.5 µM A83-01 (Tocris; 2939), 10 nM Gastrin (Sigma; G9145), 4 nM R-spondin (R&D Systems; 4645-RS-100), 4 nM Noggin (R&D Systems; 6057-NG-100), 5 nM fibroblast growth factor (R&D Systems; 345-FG-250), 5 ng ml^−1^ epidermal growth factor (R&D Systems; 236-EG-200), 3 µM p38i SB202190 (Sigma; S7067) and 10 µM Rho-kinase inhibitor Y-27632 (Sigma; Y0503).

### Bulk RNA sequencing and analysis in organoids

Organoids were treated with or without dabrafenib (100 nM) + trametinib (10 nM) or dabrafenib (100 nM) + ERAS007 (100 nM) for 72 h and subjected to RNA extraction using the RNeasy Kit (Qiagen). A strand-specific transcriptome library was constructed and sequenced at BGI Genomics using the DNBSEQ platform. Paired-end 100-base pair RNAseq, 20 million reads per sample, were mapped to the reference genome (GCF_000001405.38_GRCh38.p12) using HISAT. Bowtie2 was used to align the clean reads to the reference genes. GO enrichment analysis was performed using the GO enrichment analysis web-based platform (http://geneontology.org/docs/go-enrichment-analysis/)^39,40^. Genes with significant differential expression (log_2_(fold change) (FC) > 1; *P* < 0.05) were used for the enrichment test. The gene signature score was calculated as the mean of the log_2_ normalized expression of all genes in each gene signature. The same genes in the epithelial ISG program and the MAPK signature from scRNAseq analysis were used to calculate the ISG score and MAPK score here. Gene signature score delta was calculated as the score in treated samples subtracted by the score in untreated samples.

### Quantitative PCR

RNA extraction was performed using the RNeasy Kit (Qiagen) per the manufacturer’s protocol. Reverse transcription was performed using qScript cDNA SuperMIx (Quantabio). qPCR analysis was performed using TaqMan Gene Expression Master Mix (Thermo Fisher Scientific) on the Roche Light Cycler 480. TaqMan Gene Expression Assays of IFIT1 (Hs03027069_s1), IFIT2 (Hs01922738_s1), IFIT3 (Hs01922752_s1), IRF1 (Hs00971965_m1), CXCL9 (Hs00970538_m1), CXCL10 (Hs00171042_m1), CXCL11 (Hs00171138_m1), DUSP6 (Hs04329643_s1), ETV4 (Hs00383361_g1), ETV5 (Hs00927578_g1) and SPRY4 (Hs01935412_s1) were purchased from Thermo Fisher Scientific. B-actin (Thermo Fisher Scientific; 4326315E) was used as endogenous control.

### Western blot

ABPS cells were treated with dabrafenib (100 nM) and trametinib (10 nM) for 4, 24, 48 and 72 h (drug was refreshed every 24 h) and subjected to western blotting as previously described^9^ using antibodies to phospho-RSK1 (abcam; ab32413; 1:1,000 dilution) and GAPDH (Millipore Sigma; MAB374; 1:1,000 dilution).

### Cell viability assay

APSe and ABPS cells were seeded at concentrations of 5 × 10^3^ and 2 *×* 10^3^ cells per well, respectively, in a 96-well plate. Cells were incubated for 24 h and treated with dabrafenib (100 nM), trametinib (10 nM) or the combination of DT for 72 h. Cell viability was measured using CellTiter-Glo (Promega) according to the manufacturer’s protocol.

### RNAseq analysis from the BRAF/MEK/EGFRi combination trial

Bulk RNA sequencing data in 71 patients (including 45 paired day 1 and day 15 biopsy samples and 26 separate biopsy samples from baseline) enrolled in the previous BRAF/EGFRi ± MEKi trial with dabrafenib, panitumumab and trametinib in patients with BRAF^V600E^ CRC were obtained from Novartis^25^. RNA sequencing data were trimmed mean of M values normalized^41^. Normalized expression data were then corrected for varying levels of liver gene expression using the expression of a 22-gene score (Supplementary Table 5) in a linear model to reduce the impact of biopsy location on the expression data. All expression values are log_2_ of liver-corrected counts per million. Gene signature expression levels are the mean log_2_ of corrected counts for all genes in the signature. Genes used for gene signature score calculation are listed in Supplementary Table 5.

### Tissue processing and scRNAseq

Core needle biopsies were obtained from interventional radiology at Massachusetts General Hospital and Brigham and Women’s Hospital and transported in ice-cold hypothermosol before processing. Per patient and time point, the first two to three cores from the operative procedure were allocated for scRNAseq and cut into small pieces with scissors in a 1.5-ml Eppendorf tube containing 1 ml of enzymatic digestion mix (Miltenyi; Human Tumor Dissociation kit). The Eppendorf tubes were then transferred to a rotation shaker set to 37 °C and 550 r.p.m. and shaken for 15 min. The digestion mix was subsequently filtered through a 50-μm Celltrics strainer sitting on a 15-ml falcon tube on ice and mechanically dissociated once more with the plunger of a 1-ml syringe against the screen. The filter and enzymatic mixture were washed with RPMI containing 0.5% bovine serum. The cell suspension was spun at 1,500 r.p.m. (524*g*) for 4 min at 4 °C in a precooled centrifuge to pellet the cells. The pellet was lysed in 300 μl ammonium–chloride–potassium buffer for 2 min on ice, transferred into an 1.5-ml Eppendorf tube and then stopped with 1.1 ml RPMI containing 0.5% bovine serum. The cell suspension was then centrifuged at 1,500 r.p.m. (524*g*) for 4 min at 4 °C. The resulting cell pellet was resuspended in RPMI containing 0.5% bovine serum, filtered again through a 50-μm Celltrics strainer into a new 1.5-ml Eppendorf tube, spun at 1,500 r.p.m. (524*g*) for 4 min at 4 °C and then resuspended in 20 μl RPMI + 0.5% bovine serum. Cells were counted and loaded as 8,000 cells per channel using the 10× Genomics Single Cell 5’ Reagent Kit v.2. If cell counts permitted, up to three channels were loaded per patient and time point; 10× libraries were constructed according to the manufacturer’s instructions and sequenced at the Broad Institute Genomics Platform.

### scRNAseq preprocessing, QC filtering and clustering

CellRanger v.6.0.2 was used to align reads to the GRCh38 human genome reference and aggregate all samples into a single feature-barcode matrix. Depth normalization was turned off during the aggregation. Starting with the filtered feature-barcode matrix, cells with gene counts of <200, mitochondrial gene levels of >50% or scrublet-based doublet scores of >0.3 were filtered out, keeping ~90% of cells. Leiden clustering was performed in Scanpy and clusters were manually annotated for major cell types using canonical markers such as epithelial cell adhesion molecule for epithelial cells.

### Epithelial cell-specific QC filtering

Epithelial cells were analyzed separately from the immune and stromal cells. A cluster of epithelial cells in patient 26 was identified as small intestinal epithelial cells from the adjacent nonmalignant tissue and excluded from further analysis. A minimum threshold of 1,742 genes per cell was set based on the local minimum in the observed bimodal distribution of genes per cell to exclude cells with low gene counts that likely represent dead cells.

### Identification of DEGs

Differential gene expression analysis was performed using the FindMarkers function in the Seurat v.4.1 R package separately in responders (>6 months survival) and nonresponders (<6 months survival) for pre- versus on-treatment specimens. Since epithelial cell numbers per specimen were very variable, we randomly downsampled the cells in specimens with large cell numbers to the median cell count of 557 per specimen. The volcano plots call out all genes with Bonferroni corrected *P* values < 0.05 and |log_2_FC| ≥ 1.

### Calculating gene signature scores

We used the AddModuleScore function of the Seurat v.4.1 R package^42,43^. For each cell, this calculates the average expression of genes in the module subtracted by the average expression of a randomly selected set of control genes with similar expression across the cells. As input to the function, we used normalized expression and the default setting of 100 random control genes. Genes used to calculate signature scores were listed in Supplementary Table 4.

### Gene set enrichment analysis

We used g:Profiler (https://biit.cs.ut.ee/gprofiler/gost)^44^ for the gene set enrichment analysis. We performed an ordered query of significant upregulated genes (log_2_FC > 1, Bonferroni-corrected *P* values < 0.05) in patients with PFS > 6 months and PFS < 6 months.

### Statistical analyses

Experimental data were expressed as the mean ± s.e.m. of three or more individual experiments. The two-tailed Wilcoxon rank sum test was used to evaluate differences between unpaired data; the Wilcoxon signed rank test was used to compare paired data.

As detailed in the clinical protocol, descriptive statistics were used to summarize efficacy, including response rate (with 95% CI). PFS and median overall survival were calculated via Kaplan–Meier. Descriptive statistics were also used to report rates of AEs. General power calculations for the clinical trial cohort were based on a sample size of at least *n* = 25 providing 80% power to detect a difference in response rate of 22% compared with historical controls using a one-sided binomial test with alpha of 0.10.

### Reporting summary

Further information on research design is available in the Nature Portfolio Reporting Summary linked to this article.

## Online content

Any methods, additional references, Nature Portfolio reporting summaries, source data, extended data, supplementary information, acknowledgements, peer review information; details of author contributions and competing interests; and statements of data and code availability are available at 10.1038/s41591-022-02181-8.

### Supplementary information

Reporting Summary

Supplementary Table 1 Tumor mutational burden.

Supplementary Table 2 DEG_epithelial_On versus pretreatment in patients with PFS > 6 months and PFS < 6 months from scRNAseq analysis.

Supplementary Table 3 Gene set enrichment analysis using Gprofiler.

Supplementary Table 4 ISG and MAPK gene list used for the ISG and MAPK scores calculation.

Supplementary Table 5 RNAseq gene signatures from BRAF/MEK/EGFRi trial.

### Source data

Source Data Fig. 1 Full western blots for Extended Data Fig. 1b.

## Figures and Tables

**Extended Data Table 1:**
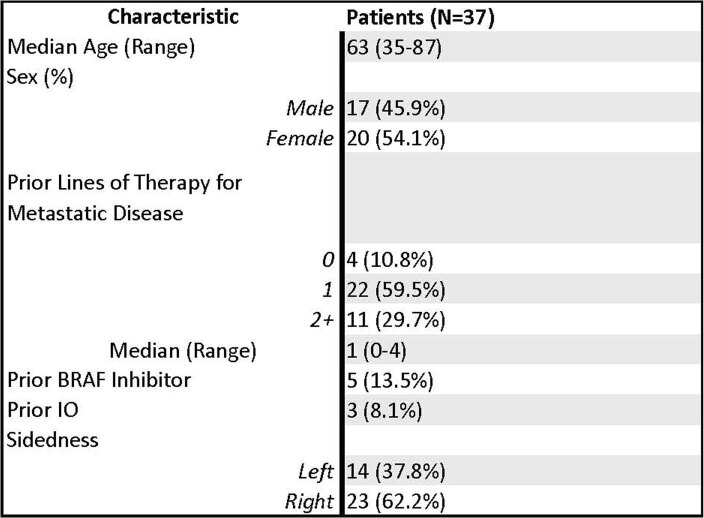
Patient demographics

Demographics of enrolled 37 patients

**Extended Data Table 2:**
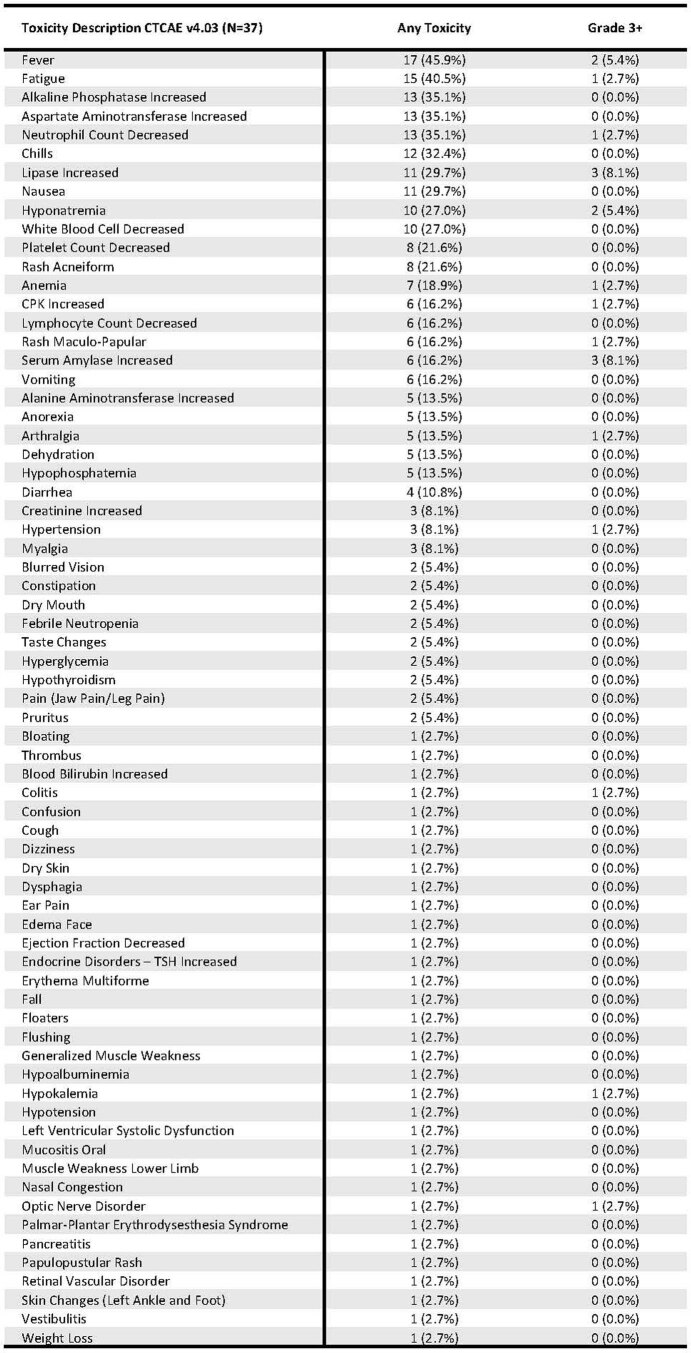
Toxicity AE summary

Toxicity and AE summary of enrolled 37 patients

## Data Availability

Complete deidentified patient data (including study protocol) will be available indefinitely within 2 years after the last patient’s last survival follow-up visit and will be uploaded to clinicaltrials.gov. Sequencing data of deidentified human subject specimens are deposited at dbGaP: phs003178. Any additional information required to reanalyze the data reported in this paper is available from the corresponding author upon request from the publication of the paper. Single-cell sequencing data is available here: https://singlecell.broadinstitute.org/single_cell/study/SCP2079/combined-pd-1-braf-and-mek-inhibition-in-braf-v600e-colorectal-cancer-a-phase-2-trial. Requests for data sharing will be responded to within 2–3 weeks. Source data are provided with this paper.

## Extended data

Extended data is available for this paper at 10.1038/s41591-022-02181-8.
