# Supplementary material for: Combined PD-1, BRAF and MEK inhibition in BRAFV600E colorectal cancer: a phase 2 trial
Source: Nat Med. 2023 Jan 26;29(2):458–66. doi: 10.1038/s41591-022-02181-8 (PMC9941044; doi:10.1038/s41591-022-02181-8)
Supplement: Supplementary file 1 — Reporting Summary [file 41591_2022_2181_MOESM1_ESM.pdf]

nature research

Corresponding author(s): Ryan Corcoran
Nir Hacohen

Last updated by author(s): Jan 11, 2023

Reporting Summary

Nature Research wishes to improve the reproducibility of the work that we publish. This form provides structure for consistency and transparency in reporting. For further information on Nature Research policies, see our Editorial Policies and the Editorial Policy Checklist.

Statistics

For all statistical analyses, confirm that the following items are present in the figure legend, table legend, main text, or Methods section.

n/a	Confirmed	
☐	☒	The exact sample size (*n*) for each experimental group/condition, given as a discrete number and unit of measurement
☐	☒	A statement on whether measurements were taken from distinct samples or whether the same sample was measured repeatedly
☐	☒	The statistical test(s) used AND whether they are one- or two-sided<br>*Only common tests should be described solely by name; describe more complex techniques in the Methods section.*
☒	☐	A description of all covariates tested
☒	☐	A description of any assumptions or corrections, such as tests of normality and adjustment for multiple comparisons
☐	☒	A full description of the statistical parameters including central tendency (e.g. means) or other basic estimates (e.g. regression coefficient) AND variation (e.g. standard deviation) or associated estimates of uncertainty (e.g. confidence intervals)
☐	☒	For null hypothesis testing, the test statistic (e.g. F, t, r) with confidence intervals, effect sizes, degrees of freedom and P value noted<br>*Give P values as exact values whenever suitable.*
☒	☐	For Bayesian analysis, information on the choice of priors and Markov chain Monte Carlo settings
☒	☐	For hierarchical and complex designs, identification of the appropriate level for tests and full reporting of outcomes
☐	☒	Estimates of effect sizes (e.g. Cohen's d, Pearson's r), indicating how they were calculated

Our web collection on statistics for biologists contains articles on many of the points above.

Software and code

Policy information about availability of computer code

Data collection	ZEN 2 (blue edition)
Data analysis	MuTect1, Strelka2, STAR, RSEM, Halo software, HISAT, Bowtie2, CellRanger version 6.0.2, Seurat v4.1

For manuscripts utilizing custom algorithms or software that are central to the research but not yet described in published literature, software must be made available to editors and reviewers. We strongly encourage code deposition in a community repository (e.g. GitHub). See the Nature Research guidelines for submitting code & software for further information.

Data

Policy information about availability of data

All manuscripts must include a data availability statement. This statement should provide the following information, where applicable:

- Accession codes, unique identifiers, or web links for publicly available datasets
- A list of figures that have associated raw data
- A description of any restrictions on data availability

Complete deidentified patient data (including study protocol) will be available indefinitely within 2 years after the last patient's last survival follow-up visit and will be uploaded to clinicaltrials.gov. Sequencing data of deidentified human subject specimens are deposited at dbGaP: phs003178. Any additional information required to reanalyze the data reported in this paper is available from the corresponding author upon request from the publication of the paper. Single-cell sequencing data is available here : https://singlecell.broadinstitute.org/single_cell/study/SCP2079/combined-pd-1-braf-and-mek-inhibition-in-braf-v600e-colorectal-cancer-a-phase-2-trial. Requests for data sharing will be responded to within 2–3 weeks. Source data are provided with this paper.

Field-specific reporting

Please select the one below that is the best fit for your research. If you are not sure, read the appropriate sections before making your selection.

- [x] Life sciences
- [ ] Behavioural & social sciences
- [ ] Ecological, evolutionary & environmental sciences

For a reference copy of the document with all sections, see nature.com/documents/nr-reporting-summary-flat.pdf

Life sciences study design

All studies must disclose on these points even when the disclosure is negative.

Sample size	37 BRAFV600E CRC patients have been enrolled in this clinical trial (NCT03668431). General power calculations for the clinical trial cohort were based on a sample size of at least n=25 providing 80% power to detect a difference in response rate of 22% compared to historical controls using a one-sided binomial test with alpha of 0.10.
Data exclusions	Patient 33 was excluded for the best percent change analysis, since this patient stopped treatment before first restaging scan was performed.
Replication	At lease 3 independent biological experiments were taken to verify the reproducibility of the experimental findings.
Randomization	Samples/organisms/participants were randomly allocated into experimental groups.
Blinding	The investigators were blinded to group allocation during data collection and analysis.

Reporting for specific materials, systems and methods

We require information from authors about some types of materials, experimental systems and methods used in many studies. Here, indicate whether each material, system or method listed is relevant to your study. If you are not sure if a list item applies to your research, read the appropriate section before selecting a response.

Materials & experimental systems

n/a	Involved in the study	
☐	☒	Antibodies
☐	☒	Eukaryotic cell lines
☒	☐	Palaeontology and archaeology
☐	☒	Animals and other organisms
☐	☒	Human research participants
☐	☒	Clinical data
☒	☐	Dual use research of concern

Methods

n/a	Involved in the study	
☒	☐	ChIP-seq
☒	☐	Flow cytometry
☒	☐	MRI-based neuroimaging

Antibodies

Antibodies used	Immunostaining Primary Antibodies:<br>phospho-RSK1 (abcam; ab32413; clone: E238)<br>GAPDH (Millipore Sigma; MAB374; clone: 6C5)<br>anti-CD3 (Abcam; ab11089; clone: CD3-12)<br>anti-CD8 (Cell signaling; 98941S; clone: D4W2Z)<br><br>Secondary Antibodies:<br>Goat anti-Rabbit IgG (H+L) Highly Cross-Adsorbed Secondary Antibody, Alexa Fluor 568 (Thermo Fisher Scientific; A-11036)<br>Goat anti-Rat IgG (H+L) Cross-Adsorbed Secondary Antibody, Alexa Fluor 488 (Thermo Fisher Scientific; A-11006)
Validation	All antibodies are commercially available and validated by the supplier. Antibodies including phospho-RSK and GAPDH were also validated in Tanaka, et al. Cancer Discovery, 2021

Eukaryotic cell lines

Policy information about cell lines

Cell line source(s)	APS cell line was generated from organoids established from colon tissue of C57BL/6 mice harboring a conditional floxed Trp53 allele and then infected with Cre-expressing adenovirus for Trp53 deletion and subjected to CRISPR/Cas9 knock-out of

Apc and Smad4. ABPS and APSe cell lines were generated from APS cell line by expressing BRAF V600E and empty vector, respectively. HEK293 cell line was purchased from ATCC.

Authentication	All cell lines were authenticated by whole exome sequencing.
Mycoplasma contamination	All cell lines tested negative for mycoplasma.
Commonly misidentified lines (See ICLAC register)	No commonly misidentified cell lines were used.

Animals and other organisms

Policy information about studies involving animals; ARRIVE guidelines recommended for reporting animal research

Laboratory animals	Mus musculus, C57BL/6J, male, age 10-12 weeks. Sex was not considered in the animal study design.
Wild animals	The study did not involve wild animals.
Field-collected samples	The study did not involve field-collected samples.
Ethics oversight	Massachusetts General Hospital IACUC approved the animal studies.

Note that full information on the approval of the study protocol must also be provided in the manuscript.

Human research participants

Policy information about studies involving human research participants

Population characteristics	37 of a planned 40 BRAFV600E CRC patients have been enrolled. All 32 slots for MSS patients have been accrued as well 5 of 8 slots reserved for MSI patients. 5 patients had prior therapy with either BRAF inhibitors and/or immune checkpoint inhibitors. Median age was 63 (range 35-87) and 20 (54.1%) were female. Sex was not considered in the study design and sex of participants was determined based on self-report. More detailed patients demographics information is listed in supplemental table S1.
Recruitment	The study was offered to all eligible patients at MGH and Dana-Farber Cancer Institute. Eligible patients must have histologically or cytologically confirmed metastatic colorectal cancer and a documented BRAF V600E mutation by a CLIA-certified laboratory test and must be wild-type for KRAS and NRAS. Patients were required to be aged ≥18 years, have measurable disease according to RECIST v1.1, have an Eastern Cooperative Oncology Group (ECOG) performance status of ≤2, and have adequate baseline organ function (as determined by laboratory parameters). The trial was amended after first 9 patients to exclude patients with prior BRAF or MEK inhibitor or immunotherapy. Key exclusion criteria included chemotherapy or radiotherapy within 4 weeks prior to entering the study, and any serious or unstable preexisting medical condition. All patients provided written informed consent before the study.
Ethics oversight	The study was conducted in accordance with Guidelines for Good Clinical Practice and the ethical principles described in the Declaration of Helsinki, and approved by the local institutional review board.

Note that full information on the approval of the study protocol must also be provided in the manuscript.

Clinical data

Policy information about clinical studies

All manuscripts should comply with the ICMJE guidelines for publication of clinical research and a completed CONSORT checklist must be included with all submissions.

Clinical trial registration	NCT03668431
Study protocol	BRAFV600E CRC patients at the Massachusetts General Hospital Cancer Center and the Dana-Farber Cancer Institute were treated with spartalizumab (PDR001) 400mg IV q28d, dabrafenib 150mg PO BID, and trametinib 2mg PO daily. Patients received study therapy until disease progression, unacceptable toxicity, death, or discontinuation for any other reason. Safety was monitored throughout the study for all patients across cohorts via physical examinations, laboratory evaluations, vital sign and weight measurements, performance status evaluations, ocular and dermatologic examinations, concomitant medication monitoring, electrocardiograms, echocardiograms, and AE monitoring (characterized and graded per Common Terminology Criteria for Adverse Events, v4.0). AEs were recorded using standard Medical Dictionary for Regulatory Activities coding. Dose interruptions, reductions, and discontinuations for all of the study drugs were monitored.<br>The primary endpoint was ORR, secondary endpoints were progression-free survival, disease control rate, duration of response, and overall survival. Anti-tumor efficacy was assessed CT or MRI at baseline and then every 8 weeks until progression or death. Response determination was based on RECIST v1.1 by the Dana Farber/Harvard Cancer Center Tumor Metrics Core. For the subset of patients who showed a confirmed CR or PR, DOR was defined as the time in weeks from the first documented evidence of CR or PR (the first response prior to confirmation) until the time of documented disease progression or death due to any cause, whichever was first. PFS was defined as the time in weeks between the first dose and the date of disease progression or death due to any cause. Finally, OS was defined as the time in weeks from the first dose of study drug until death due to any cause. PFS and time on treatment was summarized with Kaplan-Meier methodology using medians and 95% CIs (estimated using Brookmeyer-Crowley method). Fresh tumor biopsies were collected before dose (day 1) for scRNA-seq analysis and PDOs generation as well as after dose (day 15) for scRNA-seq analysis. The same tumor lesion was biopsied at baseline and at day 15. FFPE and flash frozen tumor samples were collected at day 1 for genomic and molecular analysis.

Complete de-identified patient data (including study protocol) will be available indefinitely within 2 years after the last patient's last survival follow-up visit and will be uploaded to clinicaltrials.gov.

Data collection

Since Oct 2018, 37/40 patients at the Massachusetts General Hospital Cancer Center and the Dana-Farber Cancer Institute were enrolled.

Outcomes

Among all 37 patients, confirmed RR was 24.3% (with 2 complete responses) with a disease control rate (DCR) of 70.3%, which compares favorably to the historical 7% confirmed RR of dabrafenib plus trametinib alone in BRAFV600E CRC. The median progression-free survival (PFS) was 4.3 months. Median overall survival (OS) was 13.6 months. Median duration of time on treatment was 7.4 months. Among the 32 patients without prior BRAF-directed therapy or ICB, ORR was 28.1% and DCR was 71.9%. Among 28 patients without prior BRAF-directed therapy or ICB who were also MSS, confirmed ORR was 25% and DCR was 75%, median PFS was 5 months.
