# Supplementary figures and images for: Combined PD-1, BRAF and MEK inhibition in BRAFV600E colorectal cancer: a phase 2 trial
Source: Nat Med. 2023 Jan 26;29(2):458–66. doi: 10.1038/s41591-022-02181-8 (PMC9941044; doi:10.1038/s41591-022-02181-8)

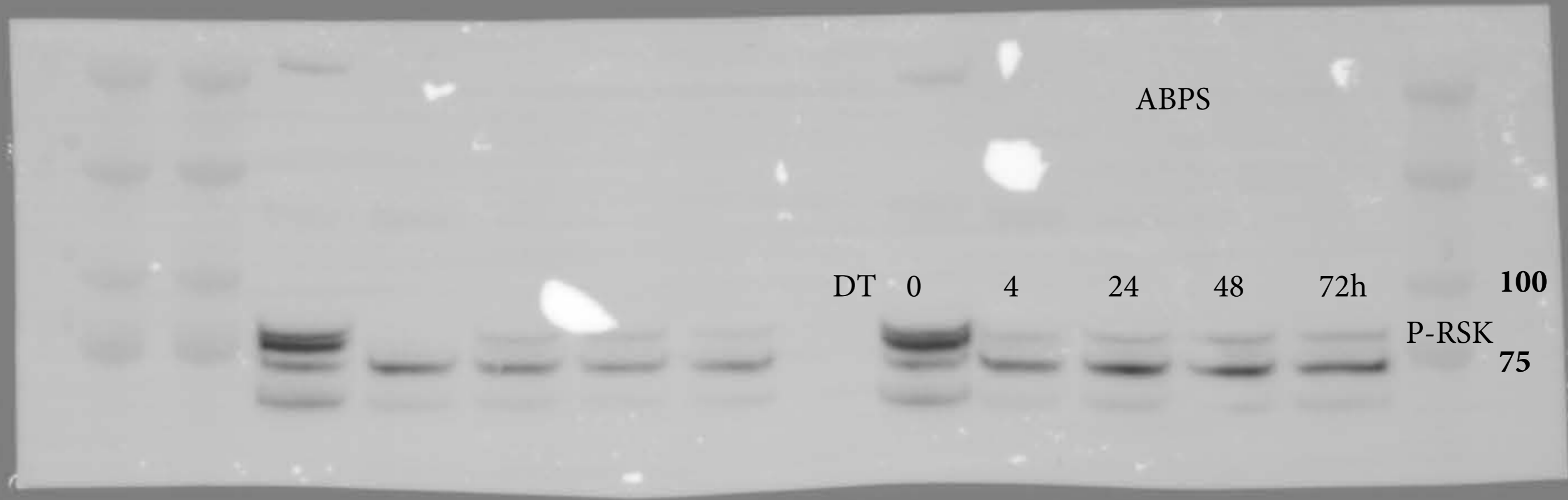

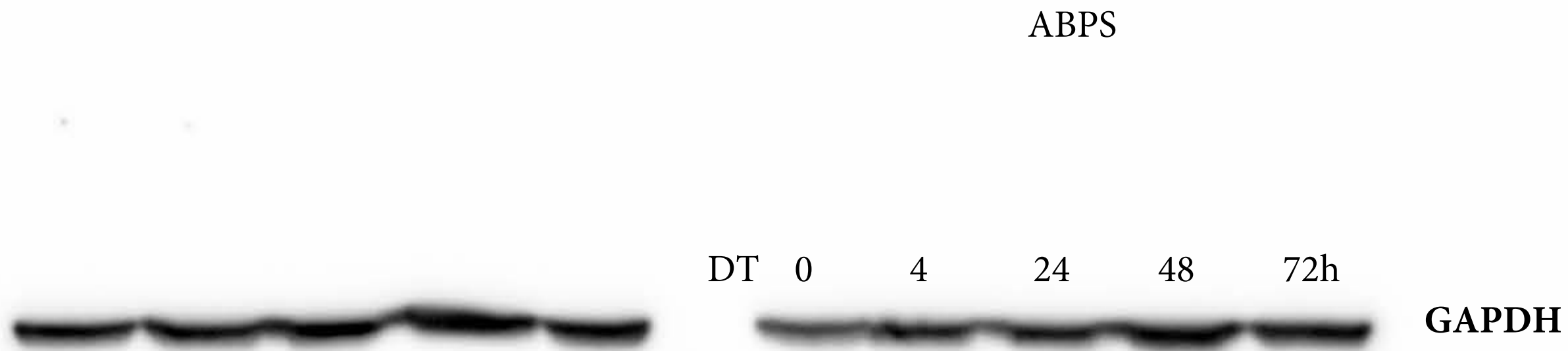

Supplement: Supplementary file 7 — Full western blots for Extended Data Fig. 1b. [file 41591_2022_2181_MOESM7_ESM.pdf]
